# Rethinking Contrast CT in the Emergency Department: Why Pretest Probability, Not Creatinine, Should Guide Our Decisions

**DOI:** 10.7759/cureus.100989

**Published:** 2026-01-07

**Authors:** So Sakamoto

**Affiliations:** 1 Department of Emergency Medicine, Asahi General Hospital, Asahi, JPN

**Keywords:** acute kidney injury, contrast-enhanced ct, diagnostic decision-making, emergency medicine, pretest probability

## Abstract

Contrast-enhanced CT plays a central role in diagnostic decision-making in the emergency department (ED). However, contrast administration is often delayed or avoided mainly based on serum creatinine levels, despite increasing evidence that the nephrotoxic risk of modern iodinated contrast agents is low. Such creatinine-centered decision-making may result in diagnostically inadequate imaging and delayed diagnosis of time-sensitive, high-risk conditions in emergency care.

This commentary examines the persistence of creatinine-centered imaging heuristics and their diagnostic consequences in emergency care, while not proposing new guidelines. Contemporary evidence shows that contrast-associated acute kidney injury (CA-AKI) is uncommon in the general ED population, with modest risk increases limited to patients with severe chronic kidney disease. In comparison, noncontrast CT is substantially less accurate for many high-risk emergency conditions, including vascular, ischemic, and infectious pathologies. Basing imaging decisions on the avoidance of contrast rather than diagnostic necessity can encourage defensive practice and undermine clinical reasoning.

CT selection in the ED should be guided primarily by pretest probability and diagnostic intent, with renal function serving as a contextual consideration rather than a gatekeeper. Refocusing imaging decisions on clinical purpose aligns practice with current evidence and supports timely, accurate diagnosis. Cultivating this reasoning skill should be recognized as a core competency in emergency medicine.

## Editorial

In emergency departments (EDs), contrast-enhanced CT is a cornerstone of rapid diagnostic evaluation. Yet in many clinical settings, contrast administration is routinely delayed or avoided until serum creatinine results are available. When renal function is perceived as “not ideal,” clinicians may opt for noncontrast-enhanced CT to avoid contrast-associated acute kidney injury (CA-AKI). This practice persists despite substantial contemporary evidence demonstrating that modern iodinated contrast agents carry far lower nephrotoxic risks than historically believed [1]. The core problem is not creatinine itself, but a misalignment between diagnostic purpose and imaging choice. The fundamental question should always be: Is intravenous contrast necessary to answer the clinical question? This is a matter of pretest probability and diagnostic necessity, not renal function alone. In time-sensitive emergency care, delaying or compromising imaging that is essential for diagnosis may pose greater harm than the risks associated with contrast administration.

This commentary does not propose a new guideline. Instead, it aims to reframe everyday CT decision-making in the ED through the lens of pretest probability and diagnostic intent, emphasizing that renal function should serve as a contextual modifier rather than a rigid gatekeeper. Creatinine-centered decision-making did not arise from negligence but from historical context. Earlier generations of clinicians were trained during an era when high-osmolality contrast agents posed a genuine nephrotoxic risk. The term “contrast-induced nephropathy” (CIN) became embedded in medical education, order sets, and institutional culture. Although the concept has been substantially revised, the terminology and associated heuristics remain [1].

In many institutions, serum creatinine effectively functions as a de facto gatekeeper for contrast-enhanced CT. This role is reinforced by institutional protocols, electronic ordering systems, and legacy guideline interpretations, rather than by contemporary evidence of nephrotoxicity. In CT-rich healthcare systems, where imaging is readily available around the clock and early diagnostic decisions are often made by junior physicians or non-emergency medicine specialists, such inherited rules spread rapidly. These heuristics are transmitted through repetition rather than re-evaluation, resulting in the propagation of errors. The language remains unchanged, even as the evidence has advanced.

Noncontrast CT is diagnostically inadequate for many high-risk emergency conditions, including aortic dissection, pulmonary embolism, mesenteric ischemia, and numerous infectious or ischemic abdominal pathologies. In these contexts, omitting contrast does not represent a conservative choice; instead, it provides incomplete information, false reassurance, and frequently necessitates repeat imaging. What appears to be caution may paradoxically increase patient harm through diagnostic delay. In emergency medicine, time itself is a diagnostic variable. Waiting for creatinine results before imaging suspected life-threatening conditions reframes the clinical problem away from disease probability and toward avoidance of a feared but uncommon complication.

Concerns regarding CA-AKI have been substantially reassessed. A landmark meta-analysis by Aycock et al., including more than 100,000 patients, found no observed association between contrast-enhanced CT and acute kidney injury, dialysis, or mortality in the general ED population [1]. Subsequent analyses have reinforced these findings, emphasizing that the nephrotoxic risk of modern low-osmolality iodinated contrast agents has been historically overestimated. More recent data provide important nuance. A 2025 evidence review by Long et al., summarizing findings from previously published systematic reviews and meta-analyses, reported no overall increase in acute kidney injury but identified a modest risk elevation among patients with severe chronic kidney disease (estimated glomerular filtration rate ≤30 mL/min/1.73 m²), with an absolute risk increase of approximately 4% [2]. This distinction is critical: the risk is real but small, and it applies to a narrowly defined subgroup. For the vast majority of ED patients, the diagnostic consequences of delaying or avoiding contrast-enhanced imaging outweigh the renal risk.

Importantly, the diagnostic penalty of noncontrast CT is no longer theoretical. In a multicenter diagnostic accuracy study using dual-energy CT, Shaish et al. demonstrated that unenhanced CT was approximately 30 percentage points less accurate than contrast-enhanced CT for evaluating acute abdominal pain in the ED [3]. False-negative and false-positive findings were common, affecting both primary diagnoses and actionable secondary findings. The authors concluded that in many patients, the risk of withholding contrast may exceed the risk of administering it [3]. Contemporary reviews further emphasize that contrast-enhanced CT has become the primary imaging modality for the evaluation of acute abdominal and vascular emergencies in modern emergency care, and that withholding contrast carries a substantial diagnostic consequence [4].

Acknowledging the safety of modern contrast media does not imply ignoring residual risks. Iodinated contrast-induced anaphylaxis, although rare, remains a clinically significant concern. In a large retrospective study, Fukushima et al. reported an incidence of approximately 0.06% per contrast administration [5]. Notably, more than half of the cases occurred in patients without identifiable risk factors, and many had previously tolerated the same contrast agent without adverse reactions. Premedication did not reliably prevent these events [5]. These findings underscore that contrast administration always carries a small but unpredictable risk. However, rarity and unpredictability do not justify defaulting to diagnostically inadequate imaging when contrast is essential to answer the clinical question. Instead, they highlight the need for preparedness and informed risk-benefit assessment without allowing fear of rare events to dominate diagnostic reasoning.

In practical terms, pretest probability shapes imaging decisions by clarifying what diagnostic information is required. For example, in patients with suspected aortic dissection, pulmonary embolism, mesenteric ischemia, or hemorrhage, contrast-enhanced CT is essential for diagnosis because vascular patency, perfusion, and active bleeding cannot be reliably assessed without contrast. Similarly, contrast substantially improves diagnostic accuracy in complicated intra-abdominal infections requiring source control, such as perforated viscus, abscess-forming infections, or ischemic bowel with secondary infection, where tissue enhancement patterns guide both diagnosis and intervention. In contrast, noncontrast CT is often appropriate when evaluating suspected renal colic, screening for intracranial hemorrhage, or confirming device or tube positioning, situations in which contrast provides limited additional diagnostic value.

As a practical bedside approach, CT decision-making can be framed around three sequential questions: (1) What diagnosis am I trying to confirm or exclude at this moment? (2) Is contrast required to answer that diagnostic question with sufficient accuracy? (3) Does the urgency of the clinical situation outweigh the potential risks of contrast administration? This structure is not intended as a checklist or algorithm, but as a cognitive scaffold to help clinicians align imaging choices with diagnostic intent, time sensitivity, and evolving clinical information.

The error lies not in considering renal function, but in allowing creatinine to function as a gatekeeper rather than a contextual modifier. Avoidance-based reasoning shifts clinical focus away from diagnostic clarity and toward defensive practice. In emergency medicine, the greater risk often lies not in contrast exposure, but in missed or delayed diagnosis. For emergency clinicians, several practical principles emerge. Serum creatinine should be a consideration, not a vetoing factor. High-risk, time-sensitive diagnoses demand imaging strategies aligned with diagnostic necessity rather than reflexive avoidance. Departments can support this approach by developing imaging pathways that prioritize clinical indication over rigid creatinine thresholds and by fostering collaboration between emergency medicine and radiology services.

Equally important is explicit teaching. Supervisors should articulate why a specific CT protocol is chosen, not merely what is ordered. In environments where imaging habits propagate rapidly, deliberate explanation helps dismantle outdated dogma and replace it with evidence-based reasoning. Such clarity supports both patient safety and the development of clinicians capable of critical diagnostic thinking. In countries such as Japan, access to advanced imaging is exceptionally high; OECD data indicate that Japan has 184 CT, MRI, and PET scanners per million population, far exceeding the OECD average of 51 [6]. In such resource-rich settings, clinicians can and should prioritize diagnostically optimal imaging strategies rather than defaulting to compromised approaches driven by outdated risk perceptions.

Emergency medicine operates at the intersection of urgency, uncertainty, and risk. Contemporary evidence demonstrates that the nephrotoxic risk of modern iodinated contrast media is limited, while the diagnostic penalty of noncontrast imaging in inappropriate contexts is substantial. Refocusing CT decisions on pretest probability aligns imaging practice with both evidence and the realities of emergency care. In many cases, the greatest risk is not contrast exposure, but missed diagnosis. Cultivating the ability to align imaging choices with diagnostic intent should be regarded as a core competency in emergency medicine, rather than a technical detail.
